# Spasticity evaluation with the Amadeo Tyromotion device in patients with hemispheric stroke

**DOI:** 10.3389/fnbot.2023.1172770

**Published:** 2023-07-05

**Authors:** Rocío Urrutia, Ane Miren Gutiérrez-Muto, Clara B. Sanz-Morère, Arantxa Gómez, Angela M. Politi, Francesca Lunardini, Marco Baccini, Francesca Cecchi, Natacha León, Antonio Oliviero, Jesús Tornero

**Affiliations:** ^1^Center for Clinical Neuroscience, Hospital Los Madroños, Madrid, Spain; ^2^Joint PhD Program in Neuroscience, University of Castilla La Mancha, Albacete, Spain; ^3^Neural Rehabilitation Group, Cajal Institute, Spanish National Research Council (CSIC), Madrid, Spain; ^4^Fondazione Don Carlo Gnocchi, Scientific Institute, Florence, Italy; ^5^Department of Experimental and Clinical Medicine, University of Florence, Florence, Italy

**Keywords:** muscle spasticity, muscle tone, rehabilitation, stroke, Amadeo, upper limb

## Abstract

**Objective:**

The objective of this study is to verify the reliability and the concurrent and discriminant validity of the measurements of spasticity offered by the robotic device, quantifying the (1) test–retest reliability, (2) correlation with the clinical evaluation using the Modified Ashworth Scale (MAS), (3) inter-rater reliability between the two physiotherapists, and (4) ability to discriminate between healthy and stroke patients.

**Methods:**

A total of 20 stroke patients and 20 healthy volunteers participated in the study. Two physical therapists (PT1 and PT2) independently evaluated the hand spasticity of stroke subjects using the MAS. Spasticity was assessed, both in healthy and stroke patients, with the Amadeo device at three increasing velocities of passive movement for three consecutive repeated assessments, while raw data of force and position were collected through an external program.

**Data analysis:**

The intraclass correlation coefficient (ICC) and the weighted kappa were computed to estimate the reliability of the Amadeo device measurements, the inter-rater reliability of MAS, and the correlation between the MAS and Amadeo device measurements. The discriminant ability of the Amadeo device was assessed by comparing the stroke and healthy subjects' spasticity measurements with the percentage of agreements with 0 in MAS for healthy subjects.

**Results:**

The test–retest reliability of the Amadeo device was high with ICC at all three velocities (ICC = 0.908, 0.958, and 0.964, respectively) but lower if analyzed with weighted kappa correlation (0.584, 0.748, and 0.749, respectively) as mean values for each velocity. The correlation between Amadeo and the clinical scale for stroke patients with weighted kappa correlation was poor (0.280 ± 0.212 for PT1 and 0.290 ± 0.155 for PT2). The inter-rater reliability of the clinical MAS was high (ICC = 0.911).

**Conclusion:**

Both MAS and Amadeo spasticity scores showed good reliability. The Amadeo scores did not show a strong clinical correlation with the MAS in stroke patients. Hitherto, Amadeo evaluation shows trends that are consistent with the characteristics of spasticity, such as an increase in spasticity as the speed of muscle stretching increases. The ability of the device to discriminate between stroke patients and healthy controls is low. Future studies adopting an instrumental gold standard for spasticity may provide further insight into the validity of these measurements.

## 1. Introduction

Spasticity is usually defined as a motor alteration or disorder characterized by an increase in the excitability of the myotatic or stretch reflex, causing an increase in tone. The speed is a determining factor, as the higher the speed, the greater the stretch-resistant reflex contraction (Thibaut et al., 2013; Wissel et al., 2015; Spasticity, 2017). This can highly interfere with movement, speech, and the patient's activities of daily living (Balci, 2018; Roman et al., 2022).

Stroke is among the neurological pathologies causing spasticity. Stroke-induced spasticity is a neurological disorder resulting from damage to the first motor neuron and can be difficult to treat in the initial periods after brain damage (Sunnerhagen, 2016). It is a complex phenomenon due to the heterogeneity of its symptoms and its effects on motor control (Sáinz-Pelayo et al., 2020). This causes patients to experience hypertonia, clonus, flexor, and extensor spasms. Spasticity presents in different forms depending on the site of the lesion, the time since lesion, and its size (Balci, 2018).

Both spasticity and muscle weakness caused by neurological damage (spastic paresis) are the most common motor disorders after stroke and markedly influence the patient, becoming a challenge during the rehabilitation process (Meseguer-Henarejos et al., 2018). Moreover, spasticity also causes other associated symptomatologies such as pain, shortening of tendons and connective tissue, contractures, decreased joint range, or further muscle weakness (Thibaut et al., 2013; Meseguer-Henarejos et al., 2018). These factors have repercussions on the rehabilitation process due to delays or changes in the treatment that alter or modify the functional recovery (Wissel et al., 2015). In addition, spasticity is related to an alteration of normal posture, which aggravates associated factors and increases fatigue, disturbs the person's sleep, and decreases the sense of safety, resulting in the need for increased medical attention and home care (Meseguer-Henarejos et al., 2018). Spasticity is often a fluctuating condition that can be exacerbated or attenuated by different factors (temperature, infection, stress, etc.) and its assessment may be difficult.

The clinical assessment process remains challenging (Balci, 2018). Traditionally, the evaluation of spasticity has been based on the application of scales, such as the Modified Ashworth Scale (MAS), Tardieu Scale, Spam Severity Scale, or Triple Spasticity Scale (TSS), among others (Balci, 2018; Sáinz-Pelayo et al., 2020). However, these existing scales are based on the clinician's perception, experience, and training over the years (Johnson, 2002). Among the measurement methods, MAS (Pandyan et al., 1999) is the most widely used to measure muscle tone and spasticity, measuring the resistance exerted by the muscle to stretching until the full range of motion (ROM) of the joint is achieved (Meseguer-Henarejos et al., 2018). Notwithstanding the spread use in clinical practice, the main limitation of this scale is that the administration velocity is not strictly determined, leading to the possibility to influence the result. Determining the degree of spasticity in an accurate and reliable way is critical and can compromise the patient's evaluation and the selection of the most appropriate rehabilitation process.

In the last years, new electromechanical devices have been developed, with a specific interest in robotic devices. Therapies using robotic devices can accelerate the process of neuroplasticity due to the constant stimulation provided by haptic interaction and the amount of proprioceptive and sensory information (Calabr et al., 2019). For instance, patients can receive timely feedback on their performance from robotic devices and achieve better adherence to treatment with an introduction of interactive games or tasks (Chien et al., 2020). In addition to provide repetitive, high-intensity training, stroke survivors can perform independent training with less supervision from therapists (Mehrholz et al., 2018; Chien et al., 2020).

Therapies using robotic devices have been implemented in rehabilitation sessions and are now recommended in several guidelines for stroke patients (Serrano-López Terradas et al., 2022). Robotic devices are a support tool for the therapist to intensify motor relearning, assist the patients according to their needs, quantify performance by providing feedback during therapy, and allow repetitive and high-intensity training (Jakob et al., 2018; Dehem et al., 2019; Aprile et al., 2020; Esquenazi et al., 2021). Robotic devices are also capable of measuring patient's performance, helping professionals by providing an objective assessment of various components of motor impairment (Keller et al., 2015; Dehem et al., 2017). This objective assessment could be used, for example, to personalize the rehabilitation treatment or adjust medication.

There are several types of robotic devices for hand treatment, such as exoskeletons and end-effector systems, all dedicated to motor rehabilitation (Calabr et al., 2019; Tyromotion, 2023). Some of these devices, due to the presence of sensors and actuators, include the possibility to assess upper limb kinematics and provide an objective and quantitative evaluation of arm movements after brain damage (Dehem et al., 2017). Some devices such as the REAplan robot (Dehem et al., 2017), Reharob system, HWARD, Reogo (De-la-Torre et al., 2021), MIT Manus (Bosecker et al., 2010), HapticKnob (Lambercy et al., 2010), and Tyromotion Amadeo device (Tyromotion, 2023) aim also to assess and grade the spasticity. We will focus on the Amadeo Tyromotion robotic device due to its ability to provide an automatic spasticity assessment of the hand and the individual fingers in stroke patients.

Amadeo Tyromotion is a robotic device oriented to motor and sensory rehabilitation of the hand that also allows the assessment of spasticity. It contains several programs designed for any stage of a hand affected by a neurological pathology (Butt et al., 2020). It consists of a screen facing the user who can interact with the robot through games, tasks, or more specific programs in an interactive format. The hand and forehand are placed on a platform that is connected to the main unit. The wrist is restrained with a Velcro band to prevent movement of the elbow and shoulder. The fingers are attached through magnets to the unit's rails. The visual feedback on the screen is an added factor for functional motor rehabilitation. Amadeo can be adapted to any type of patient, whether adult or pediatric, offering therapeutic exercises through games aimed at motor control during grip functions (finger flexion) or hand release (finger extension) (Fasoli and Adans-Dester, 2019). The robot also quantifies the measuring tone, spasticity, strength, and ROM through games and specific tests, monitoring the status and progress of patients using the device (Germanotta et al., 2020).

The aim of this study is to describe the quality of the spasticity measures provided by Amadeo Tyromotion and compare it with the clinical assessment. We aim to verify the reliability, concurrent and discriminant validity of spasticity measurements offered by the robotic device.

## 2. Materials and methods

### 2.1. Material

**Figure 1:**
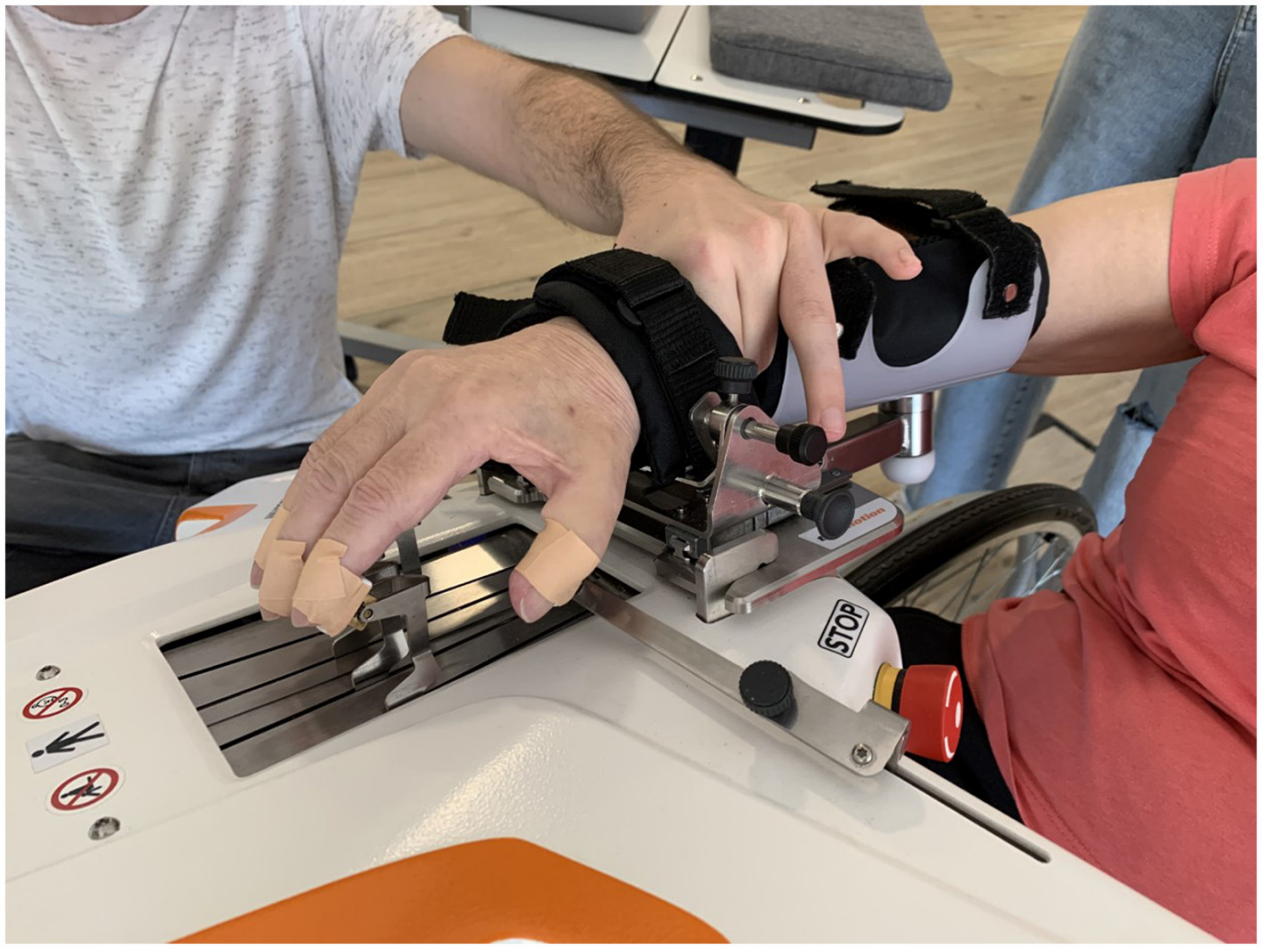
Amadeo Assessment of MAS scale with the hand support of the physical therapist while clinical evaluation.

For this study, we used the Amadeo Tyromotion robotic device (Tyromotion, 2023), a robot for hand rehabilitation that includes both motor and sensory rehabilitative components (see Figure 1). To ensure that the data acquisition was accurate, the hand unit (i.e., a main platform where force and position sensors are located) was sent for calibration to Tyromotion's factory.

To perform the Amadeo Tyromotion therapy, the PT places the forearm of the patient's affected limb on a platform, restraining the arm and wrist with straps to ensure the stability of the limb on the device (see Figure 1). Then, each finger is independently placed on magnetized rails, directly coupling the person with the device, which allows the flexion–extension movement of the fingers. The Amadeo has three operation modes of mobility treatment as follows: passive, active assisted, and active movement. The robot is able to calibrate the full passive range of motion for each finger before the start of a session and provides assistive force to complete the remaining ROM during the exercise. In addition, the maximum flexion and extension force of each finger is recorded to calibrate the exercise when force control is needed. Amadeo also provides automatic measures of ROM, strength, muscle tone, and spasticity (Bishop et al., 2017).

The assessment and quantification of spasticity are based on algorithms that calculate, from raw data, both MAS and Tardieu scale, attributing a score for each finger and the full hand. In the current study, we focus only on the MAS assessment, since it is most often used clinically in the assessment of adults. The robot starts from a position of finger flexion, and it extends the fingers in two different groups as follows: the thumb (finger one) on one side in a separate way and the other fingers (from finger two to finger five) on the other side. The Amadeo selects a time window during finger extension, in which it performs the spasticity measurement (0–100% ROM, individually for each finger). If an unexpected finger flexion reaction is detected during the evaluation and the finger cannot reach the full ROM, the finger slide automatically stops, and the spasticity evaluation finishes, flexing the fingers again to end the assessment, attributing the corresponding degree of spasticity based on the force exerted against that extension. During the Amadeo spasticity assessment, the fingers are extended at three different velocities as follows: slow (v1 ≈ 0.01 m/s), medium (v2 ≈ 0.05 m/s), and fast (v3 ≈ 0.1 m/s). Although the MAS is performed clinically at a single speed, here, we retained the Amadeo spasticity measurements at the three velocities, to test the effect of speed on the assessment.

For data collection, data acquisition software is used. This program records the force and position data of the fingers in real time, while the subject is performing the therapy with the Amadeo device. After collecting data, these were processed with Matlab (R2021a, The MathWorks Inc.).

Physical therapists used the MAS to assess the patients' hand spasticity at a single high speed, as established by the scale (UAB UA, 2014).

### 2.2. Participants

In this study, we analyzed data obtained from 40 volunteer participants, recruited through the Hospital Los Madroños (Madrid), divided into two groups:

1. The control group, composed of 20 healthy subjects, was selected based on the inclusion criteria as follows: (1) being aged between 18 and 80 years (2), their age being close to the mean age of the experimental group, and (3) acceptance of informed consent. The exclusion criteria for this group were as follows: (1) having previously suffered neurological pathologies, (2) presenting pathologies affecting the mobility and strength of the upper limb, (3) photosensitive epilepsy, (4) rejection of new technologies, and (5) cognitive deficits preventing them from understanding the program.
2. The experimental group was composed of 20 stroke patients from the advanced neurorehabilitation unit of hospital los madroños. the inclusion criteria for this group were as follows: (1) diagnosis of hemispheric stroke with upper limb involvement; (2) being aged between 18 and 80 years; (3) ability to provide informed consent; (4) sufficient trunk control to maintain prolonged sitting for at least the minimum time necessary to perform the robotic therapy; (5) preserved vision; (6) patient conscious and able to understand verbal commands and instructions; and (7) patients with no other concomitant pathologies affecting motor and/or sensory function. The exclusion criteria were as follows: (1) hemiparesis caused by other diagnoses; (2) pregnancy or lactation; (3) photosensitive epilepsy; (4) severe medical or psychiatric disorder; and (5) refusal of new technologies.

All participants gave their informed consent; the procedures had the approval of the institutional ethics committee (Hospital Universitario Severo Ochoa de Leganés Ethical Committee for clinical research) and were conducted in accordance with the Declaration of Helsinki.

### 2.3. Experimental design

Each participant was independently assessed on the same day by two physical therapists (PT1 and PT2) who scored the spasticity of the full hand and each finger using the MAS. The therapists were mutually blind to each other's assessment. To minimize the modulation of muscle tone induced by mobilization, the assessors were asked to estimate spasticity in <5 repetitions. Then, the participant was assessed using the Amadeo device. The positioning on the device was carried out according to the indications in the user manual provided by Tyromotion for the correct use of Amadeo. The subjects were held in a seated position, in a comfortable and relaxed posture, in a chair with backrest and armrests, and with the forearm resting on the device in pronation. The straps were adjusted to the arm and wrist, and the magnets were placed on the distal phalanx of the fingers, leaving the distal interphalangeal joint free. The experimental room was set between 21°C and 23°C, according to regulatory bodies in Spain.

**Figure 2:**
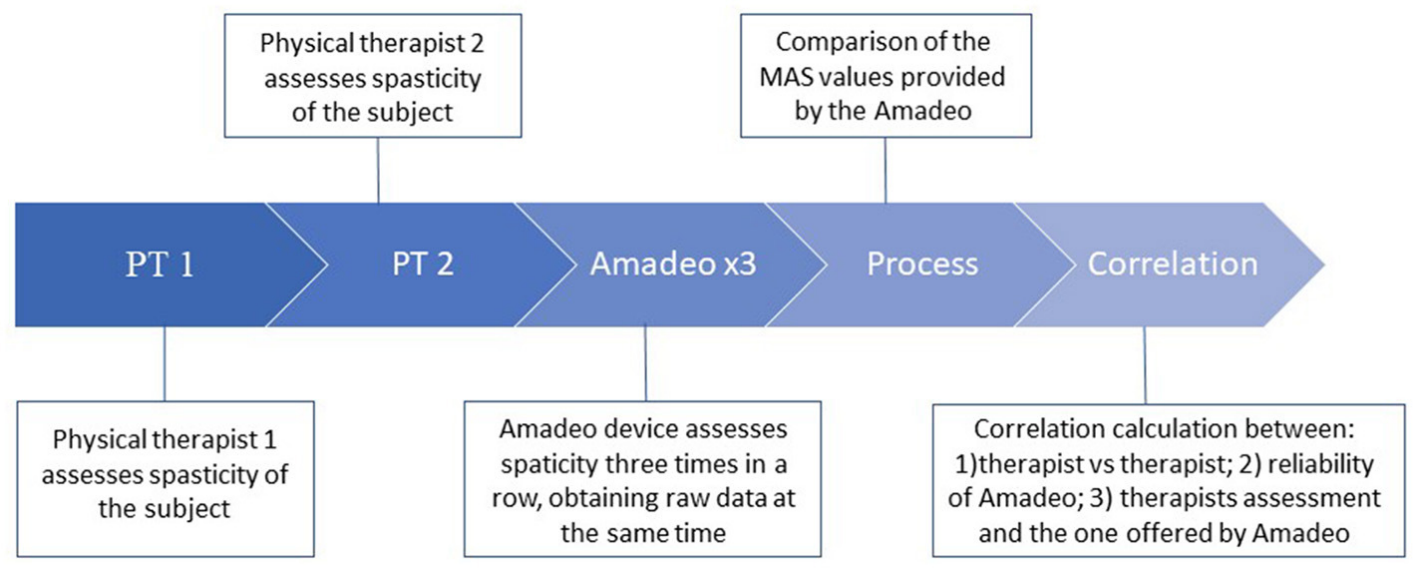
Scheme of the protocol used for the data acquisition of spasticity in subjects.

Before starting the assessment, Amadeo needs a reference of the passive ROM of each finger to establish the limits of movement in which the device will move the subject's fingers during the session. The therapist passively opened the participant's hand coupled to the device until reaching the limit of flexion–extension movements. Then, for each velocity, the Amadeo device performs a cycle consisting of the extension of fingers, maintenance of this extension, and flexion (one cycle for velocity). Amadeo provides spasticity estimation and delivers a score for both the full hand and each finger, that is assumed to be equivalent to the MAS. Raw data of position and force were acquired while spasticity assessment saved in an external device. The result of Amadeo's evaluation for each finger and the full hand was recorded in the data collection notebook at the end of the test, together with relevant observations, if applicable. The time-course of the whole evaluation is presented in Figure 2.

The three velocities mentioned above were measured, starting with the slowest (v1) and ending with the fastest (v3). At each velocity, the group from finger two to finger five was recorded first, followed by the evaluation of finger one. This procedure was repeated three times (r1, r2, and r3) (see Figure 2).

The PTs carried out the assessment in succession with ~1-min rest between the two evaluations. 1-min rest was also assured before the assessment by Amadeo.

### 2.4. Data presentation and analysis

The raw data were recorded by the software with a 200 Hz sampling rate. These were analyzed automatically by MATLAB for their interpretation. To verify whether the number of repetitions of both the PTs and Amadeo affected the evaluation, we analyzed the time-course of the MAS ratings using a Friedman test.

Amadeo's quality of data used for MAS estimation was descriptively reported in the result section. Statistical analysis was made for (1) Amadeo reproducibility assessment, (2) correlation between Amadeo and each PT evaluation, (3) correlation between clinical assessment obtained from the two PTs, and (4) capability of the Amadeo to distinguish between healthy and stroke patients. The Amadeo reproducibility assessment was obtained by correlating each velocity (v1, v2, and v3) over the three runs of evaluation (run 1, run 2, and run 3) of the whole hand using the weighted kappa coefficient.

Moreover, we also computed the one-way random effect interclass correlation coefficient (ICC), with a 95% confidence interval (Lee et al., 1989; Meseguer-Henarejos et al., 2018), Spearman (Brashear et al., 2002; de Raadt et al., 2021), and Kendall's tau correlation. We decided to use these correlation analyses, in order to compare our results with previous studies (Mokkink et al., 2020). However, we considered the kappa statistics as the more appropriate due to the characteristics of the measured variable (MAS) (McHugh, 2012).

The comparison of spasticity measured by PTs and the Amadeo device was performed using both whole hand spasticity assessment and values for each individual finger. We observed that the total values attributed by the Amadeo device in the MAS correspond to the highest spasticity value found in fingers two, three, four, and five, leaving the spasticity value given to the finger one isolated and without considering it for the MAS total scale (probably due to the bad quality of the results obtained from finger one, see “Results”). For this reason, the position and force curves for the finger one have been excluded from the data analysis.

To evaluate the capability of Amadeo to distinguish between healthy and stroke subjects, we also calculated the percentage of agreement between PTs and between Amadeo and PTs. We considered the value as correct assigned by Amadeo which agrees with the value offered by the therapists. For this purpose, the percentage of success between the Amadeo and PTs for each velocity in each run was calculated. Two variables about the agreement were calculated as follows: (i) the percentage of absolute agreement (only the exact value is considered between the Amadeo and PT); (ii) the percentage of agreement by considering agreement values that oscillate between ±1 of the MAS (i.e., with a value of MAS 1+, the values 1 and 2 are also taken as a hit, with all the values of the scale).

## 3. Results

All participants completed the whole examination. Clinical and demographic data are presented in Table 1. Full patients' characteristics and spasticity assessment scores are provided as Supplementary material.

**Table 1:** Demographic and clinical characteristics of the evaluated individuals.

**Subjects**	**Age (years)**	**Sex**	**Type of stroke**	**Time since stroke (months)**	**NIHSS**
Stroke *N* = 20	Mean: 62.5 ± 14.5 Range: 48 ± 29	Male = 10 Female = 10	Ischemic = 13 Hemorrhagic = 7	Mean: 6.1 ± 6.7 Range: 16.5 ± 14.5	Median: 10 Range: 15 ± 10
Controls *N =* 20	Mean: 53 ± 14.8 Range: 50.5 ± 28.5	Male = 7 Female = 13			

**Figure 3:**
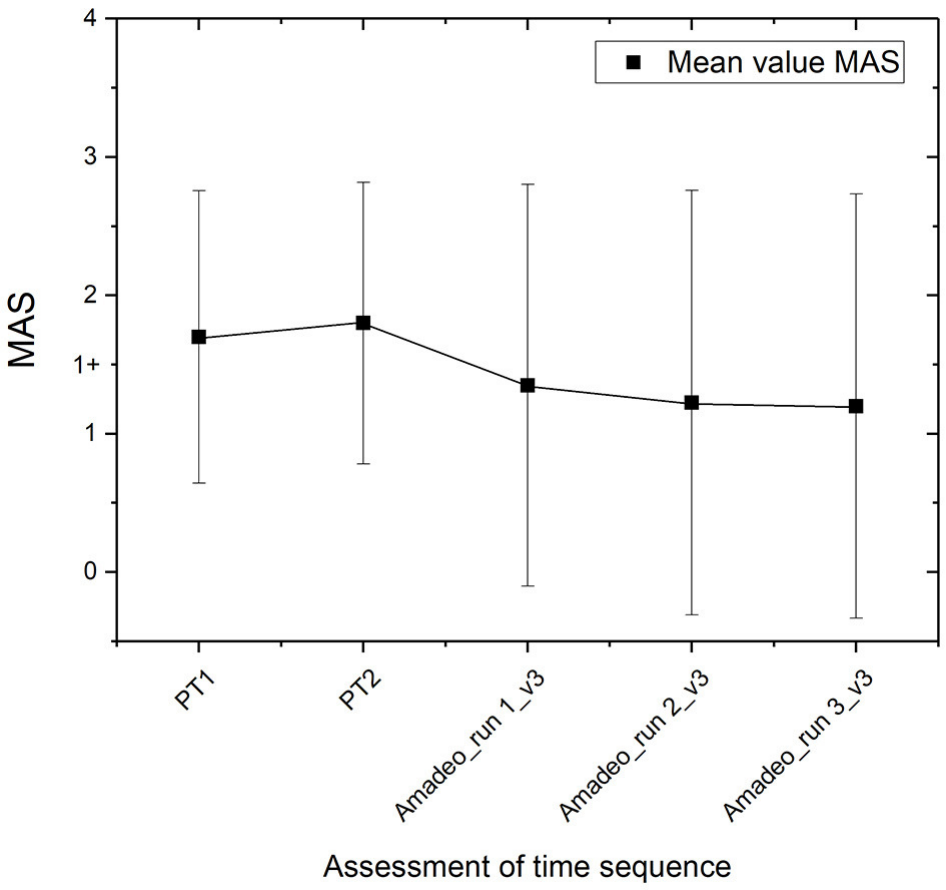
Mean MAS estimation and standard deviations of stroke patients during all the protocols of assessment.

To confirm if the number of repetitions of the PTs and Amadeo affects our evaluation, we analyzed the time-course of the MAS ratings. Figure 3 shows the mean MAS estimation and the standard deviations of all stroke patients evaluated by PTs and Amadeo in time sequence, as shown in Figure 2, including only the highest velocity, i.e., v3. No statistically significant differences among evaluations were observed (Friedman, *p* > 0.05).

### 3.1. Amadeo quality of data used for MAS estimation (descriptive)

**Figure 4:**
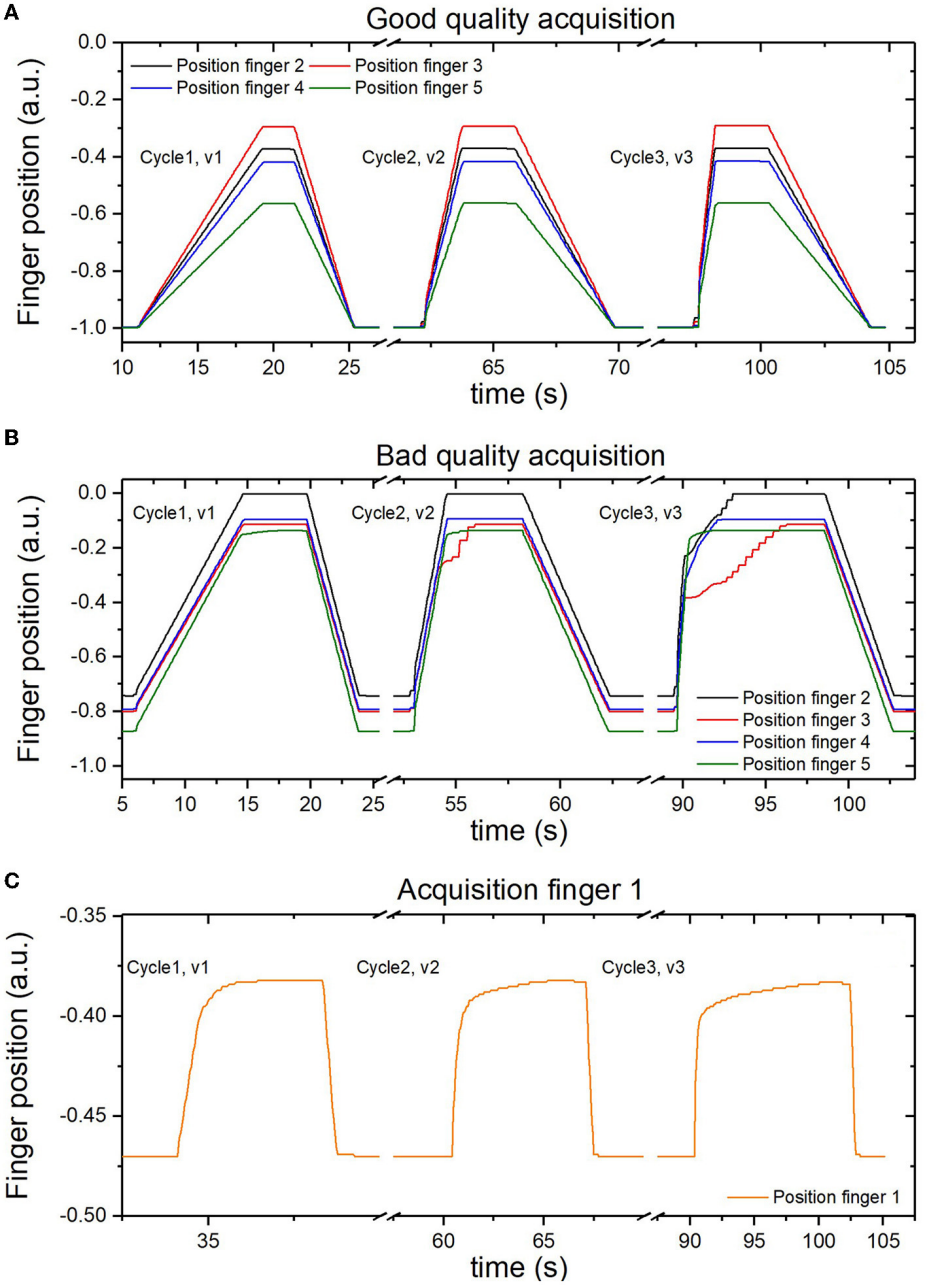
Examples of the acquired data of finger position. **(A)** with a constant velocity of extension from finger two to finger five. **(B)** with inconsistencies during the extension from finger two to finger five. **(C)** with inconsistencies found in finger one.

Figure 4 shows some examples of the finger position data from the Amadeo device during acquisitions at velocities that are assumed constant. Figures 4A, B show examples of data acquisition on the displacement from finger two to finger five. Figure 4A shows the change in the position represented by a constant velocity with corresponding to a linear performance, which we refer to as a good-quality acquisition. However, Figure 4B, shows not perfectly straight lines, for this subject at v3, which we refer to as poor-quality acquisition. Figure 4C shows examples of particularly low-quality finger one displacement. These inconsistencies occur during finger extension, i.e., the motion whose data are used by the device for spasticity assessment and are present in both healthy and stroke subjects. This can lead to errors in data analysis, resulting in incorrect assessment of spasticity. These inconsistencies are mostly found in the finger one, where the occasions, when a curve with straight lines is obtained, are rare.

**Figure 5:**
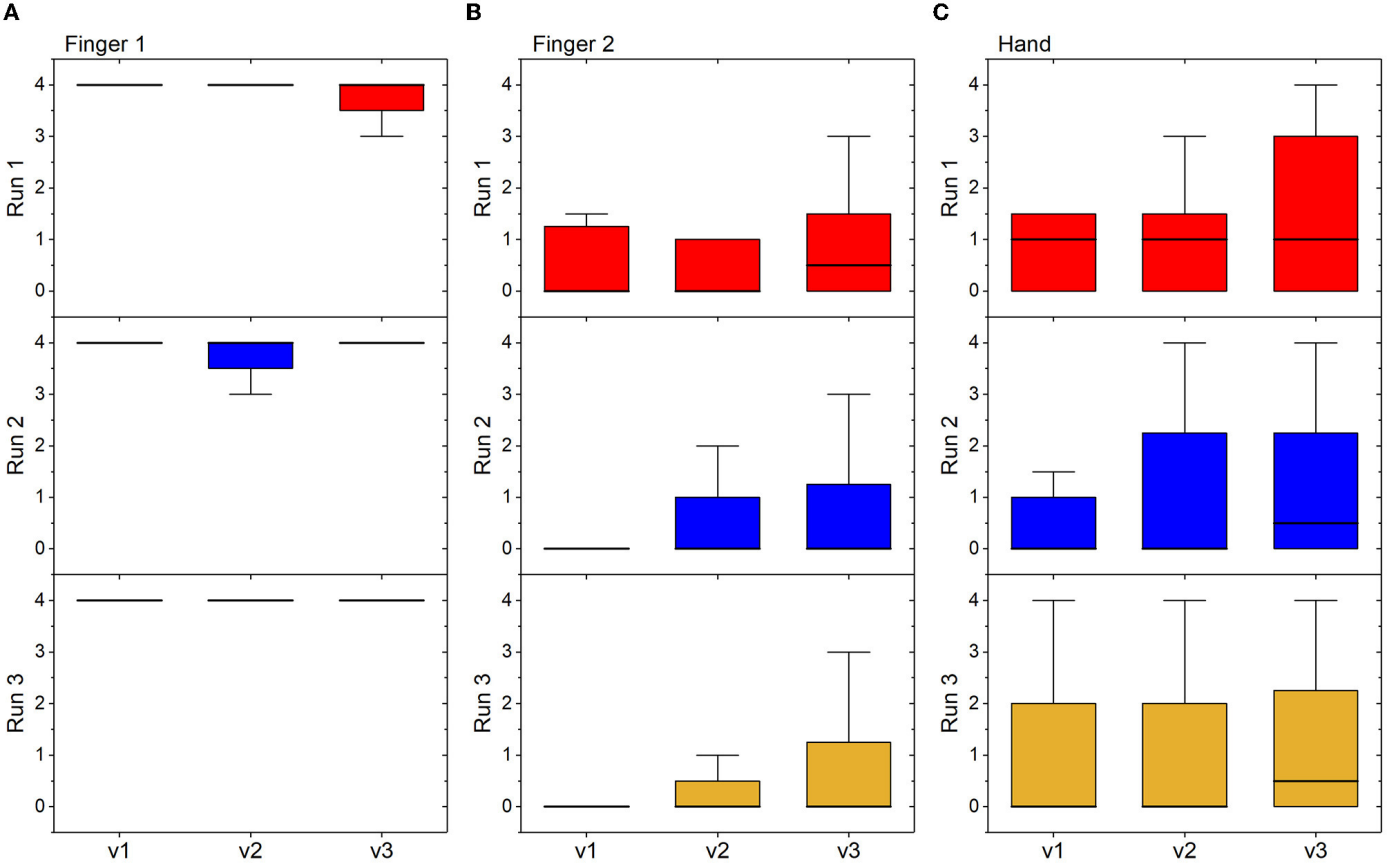
Box plots showing median, interquartile range, and minimum and maximum values, measured in the group of stroke subjects at each velocity and in each run of acquisition. **(A)** Spasticity measurements of Amadeo from finger one; **(B)** spasticity measurements of Amadeo from finger two; **(C)** spasticity measurements of Amadeo from the whole hand.

To confirm the quality of the recorded data and speed-dependency of the spasticity assessment, Amadeo's MAS estimation vs. velocity was performed. As observed in Figure 5A, finger one presents low-quality data as its value does not seem to increase with velocity: it basically presents a value of 4 on the spasticity assessed by Amadeo for any velocity in each run of acquisition for stroke patients. On the other hand, Figures 5B, C show an increase in the spasticity measurements of Amadeo with the velocity for finger two and the whole hand. This behavior was identified for the rest of the fingers.

### 3.2. Comparisons of spasticity scores obtained by PTs and amadeo

#### 3.2.1. Correlation analysis

The reproducibility of the Amadeo spasticity estimation using weighted kappa was 0.584 for v1, 0.748 for v2, and 0.749 for v3. These scores indicate a substantial agreement at least for data obtained at v2 and v3 (Bohannon and Smith, 1987). These values are presented as mean values of the three runs for each velocity, with errors of 0.064, 0.159, and 0.053, respectively. Similar good agreement was obtained also using ICC, where the results show a correlation of 0.908 for v1, 0.958 for v2, and 0.964 for v3, for the whole hand evaluation.

When correlating the Amadeo data of the whole hand with the PT evaluations, maximum correlation results were obtained at the highest velocities (v3) for both the whole hand and all fingers and ICC and weighted kappa. For example, the ICC was 0.76 with PT1 (run 1 v3, being the best correlation) and 0.72 with PT2 (run2, v3 being the best correlation). On the other hand, the worst correlations were obtained with lower velocity (v1). For example, the ICC was 0.27 with PT1 (run 1 v1 being the worst correlation) and 0.18 with PT2 (run 2, v1 being the worst correlation).

**Figure 6:**
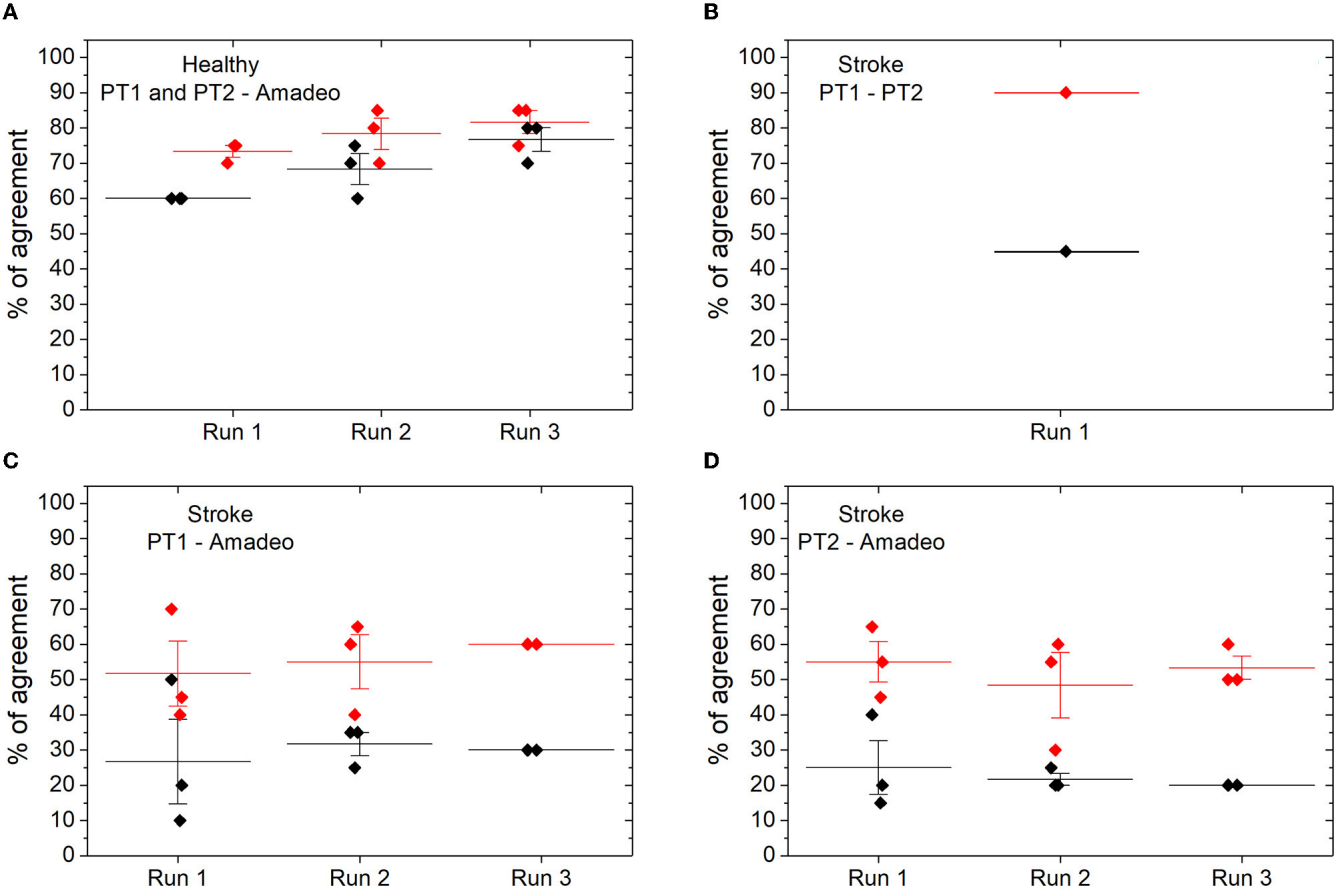
Percentage of agreements in the MAS spasticity assessment between the Amadeo device and therapists. **(A)** In relation to healthy subjects; **(B)** agreements between therapists in stroke patients; **(C)** agreements between the Amadeo device and therapist 1; **(D)** agreements between the Amadeo device and therapist 2. The percentage of absolute hits is shown in black; the percentage of absolute agreements is shown in red, and ±1 in the MAS value.

MAS evaluations between therapists (PT1 vs. PT2) showed high correlations for the hand assessment (ICC = 0.911; weighted kappa = 0.586). When individual fingers were evaluated, a satisfactory result was obtained (ICC = 0.961, Spearman's correlation = 0.867, Kendall's tau = 0.847), these data being the highest correlations found in the analysis of each finger. The percentage of agreement between PTs was also very high (see Figure 6B).

As described above, some curves obtained by Amadeo were of poor quality (inconsistencies). However, when the analysis was conducted after removing the curves with inconsistencies, the results were similar. No relationship was observed between the quality of the biomechanical curves and the agreement between the PTs and Amadeo assessment.

#### 3.2.2. Percentage of agreement

In this analysis, we considered as correct the value assigned by Amadeo that agrees with the value offered by the therapists. For this purpose, the percentage of success between the Amadeo and therapists for each velocity in each run was calculated (see Figure 6). In healthy subjects, the Amadeo device has a mean accuracy (i.e., percentage of zero values on the MAS) of 60% in run 1; 68% in run 2, and 76% in run 3, as can be observed in Figure 6A. However, if we also consider as correct MAS values of ±1 (see methods), the accuracy percentage in healthy subjects rises to 73% in run 1, 78% in run 2, and 82% in run 3 (see Figure 6A). In stroke patients, the percentage of hits between PTs, that is, the number of times that therapists rate stroke subjects with the same MAS value, we obtain a 45% hit rate when they obtain the same score, and it increases to 90% if we consider a ±1 difference in the scale between them (see Figure 6B).

As for the spasticity assessment in stroke patients, the percentage of agreement between the Amadeo and the PTs scores (i.e., same value for Amadeo and PT) was 26% for run 1, 31% for run 2, and 30% for run 3, when considering PT1 (see Figure 6C), and 25% for run 1, 22% for run 2, and 20% for run 3, when considering PT2 (see Figure 6D). If we include in the percentage of agreement also differences between the Amadeo and the PT MAS score of ±1, then this percentage increases, obtaining values between 24% and 30% for PT1, and between 26% and 33% for PT2.

## 4. Discussion

Our data can be summarized as follows: (1) the reproducibility of the Amadeo grading is high (substantial agreement obtained using weighted kappa calculation); (2) the correlation between Amadeo and PTs is higher when Amadeo evaluates spasticity at high velocity (v3), while it is very low at lower velocities (v1 and v2); (3) the reproducibility of the PT grading is high; and (4) the percentage of agreement between Amadeo and PTs is lower than the agreement between PTs. Moreover, we observed that Amadeo evaluation of the spasticity of finger one is often of very poor quality, with many inconsistencies and the absence of an observed correlation between spasticity scores and velocity. Thus, finger one should be discarded from the Amadeo spasticity assessment. The other fingers also have occasional inconsistencies, but the elimination of inconsistencies does not improve the correlation between Amadeo and PTs.

The Amadeo spasticity assessment returned the best results, both in terms of reproducibility and correlation with clinical scores, for v3. This is not surprising since, among the three speeds, v3 (0.1 m/s) is the closest one to the velocity that should be adopted in the clinical test, according to Bohannon and Smith (1987) (full ROM in 1 s) (UAB UA, 2014).

Early finger extension capacity after stroke is a critical motor sign of recovery. This capacity can be used for direct therapy to those who will most benefit from it (Orihuela-Espina et al., 2016). Quantifying spasticity, which directly influences the hand opening function, may help clinicians to identify the focus of treatment for people affected by stroke. In addition, a reliable assessment of spasticity will provide relevant and objective information about the neurorehabilitation treatment (Balci, 2018).

MAS is a simple and quick method of assessment, which does not require any equipment (Meseguer-Henarejos et al., 2018). Despite this, it is still a controversial tool as it partly depends on the person performing the assessment. Since spasticity depends on the speed of stretching, differences between raters in the velocity of passive motion may contribute to disagreement in MAS scoring (Balci, 2018). Our data confirmed partially that the operator dependency of the MAS as the percentage of the agreement to provide the exact values of MAS is low (~50%). On the other hand, when we consider as an acceptable agreement a difference of one point of the MAS, this percentage of agreement is quite high (around 90%).

The MAS inter-rater reliability (PT1 VS PT2) for the hand spasticity assessment reported a moderate-substantial weighted kappa of 0.586, and an ICC of 0.911. According to Hager (2003), ICC scores should be considered poor when they are below 0.4, sufficient if ranging between 0.4 and 0.59, good if ranging between 0.6 and 0.75, and excellent if above 0.75. Based on this, the inter-rater reliability of the two PTs in rating the spasticity of the whole hand was excellent, and even higher than what reported for other joints (Bohannon and Smith, 1987).

We also found good reliability for the robotic device, with ICC > 0.900 at all three velocities and weighted kappa >0.6 at v2 and v3. These values are much higher than those reported by Germanotta et al. (2020), who found a low reproducibility (ICC < 0.5) of the Amadeo spasticity measurements at v1 and v3. However, the study by Germanotta et al. (2020) has an important difference from the present study because it compared Amadeo evaluations performed on consecutive days, using the passive ROM of the first day. This may greatly influence the measurement, given the characteristics of spasticity and a possible different positioning of the patient on the device. Since patient positioning and passive range of motion setting are part of the Amadeo assessment procedure, the reliability of Amadeo spasticity measurements in the present study might be partially overestimated. Taking into account the characteristics of spasticity, the MAS value depends on the afferences received by the muscle spindle, so a good position is essential to avoid triggering this neural hyperreactivity and, in turn, get the spasticity assessment (Aloraini et al., 2015). Some articles have elaborated a treatment protocol with the Amadeo in which they specify the modes and times of treatment (Aprile et al., 2020), but there is no clear agreement on how the patient should be placed in the Amadeo. This issue could be very important to improve the measure and the effectiveness of the device, improving the quality of the session (Bevan et al., 2021; Meyer et al., 2021). Indeed, future studies should provide more insight into the usability of this device (Orekhov et al., 2021) in comparison to other devices (Park et al., 2020).

In the study by Esquenazi et al. (2018), the validity of the Amadeo system in measuring spasticity in stroke patients on the MAS was tested in comparison to a physical therapist, and perfect reliability was found between the two measurements (ICC = 1.0). However, the reliability was estimated by computing the average measures of ICC, that is, usually much higher than the single measurements of ICC. This finding has not been confirmed by Germanotta et al. (2020) in a multicenter trial that enrolled both stroke patients and healthy subjects, where the correlation between the MAS measured with Amadeo and the MAS measured clinically was found very low as in the present study. On the other hand, they concluded a good discriminant validity of Amadeo, with all spasticity measurements obtained from stroke patients who were statistically different from those of healthy controls. Our data do not show that the device always differentiates between these subjects, but it reaches 80% of success in the detection of healthy subjects at v3. It is important to point out that, although we were expecting a zero MAS score for healthy participants, Amadeo returned 33% of values (i.e., 353 out of 1080) above zero. However, since we did not set criteria for the setting of the flexion–extension passive ROM in the Amadeo, it is possible that inconsistent results may at least be partially related to this factor. Indeed, if the range is too wide when approaching the last degrees of movement, the device exerts a longitudinal pull on the fingers rather than a joint extension. Further research should address this issue.

This study has some limitations. First, PTs and Amadeo evaluation were performed sequentially during a single session of assessment. As we know that training may affect spasticity, it is possible that the real evaluation conditions were not the same. To avoid interfering with the reproducibility of the scale, some authors suggest that MAS should not be repeated more than five times in each record (de Raadt et al., 2021). This is because muscle tone can be modulated and has an impact on the subsequent rating of spasticity. Other authors leave some time of rest between evaluations, to avoid this interference (Mokkink et al., 2020). Our therapists carried out the assessment consecutively with 1-min rest between evaluations, and the repetitions on each finger were always <5 in each patient. The preparation phase for the Amadeo assessment (e.g., placing the magnetized guides on the patient's fingers, positioning, and immobilizing the arm and wrist on the device, setting the passive range of motion for evaluation) started 1 minute after the second PT assessment and lasted several minutes. After that, the Amadeo MAS assessment was conducted. Thus, it is unlikely that the previous evaluations have influenced the Amadeo measurements. Although we cannot exclude the fact that the absence of a longer period of rest among the evaluations may have contributed to some extent to the observed disagreement, the effect should have been minimal, if any (see Figure 3). In our opinion, this limitation cannot explain the very low percentage of agreement between PTs and Amadeo.

Another limitation is that the three acquisitions with the Amadeo device were performed in succession, without repositioning the patient's arm and hand. We chose this procedure because we were interested in studying the reproducibility of the Amadeo measurements during passive finger extension, avoiding the influence of other potential sources of variability. Of course, both arm positioning and ROM setting depend on the rater and have a great impact on the measurements, so they could greatly increase variability.

Moreover, the whole evaluation was performed on the same day, and no longitudinal study was performed. Considering that assessment of spasticity usually requires multiple longitudinal measurements, future studies should confirm our data in a longitudinal way and possibly on a larger sample of patients. Future studies should also include a more accurate standardization of the position of the hand and fingers and the ROM setting, together with stronger control over parameters such as the temperature of the environment and the time elapsed since the pathological event, to reduce errors during acquisitions. Finally, we chose the MAS as the gold standard, but the validity and reproducibility over time of this scale have been thoroughly questioned. Possibly, the Amadeo scores and the clinical MAS scores should be compared with instrumental measurements of spasticity, providing a more reliable and valid reference standard. Moreover, since the device offers an assessment also on the Tardieu scale, future studies should verify the reliability and the concurrent and discriminant validity of the measurements of spasticity offered by the robotic device for this scale. Indeed, while the MAS focuses on the resistance of the muscle to stretching, the Tardieu scale is based on the velocity exerted during stretching. It is possible that the three speeds of assessment proposed by the Amadeo protocol are therefore specifically designed for the Tardieu-like assessment, possibly returning better results.

## 5. Conclusion

In conclusion, both the clinical and the Amadeo MAS scores were reproducible, although further studies are needed to test reproducibility over different days. However, the Amadeo MAS scores did not show a strong clinical correlation with the MAS in stroke patients. This may suggest that some aspects of spasticity are engaged by Amadeo and not by the PTs and/or vice versa. It is possible that Amadeo scores related to finger 2–5 at high velocities measure a performance consistent with spasticity, but this should be verified by further research. Future studies, including standardization of the position of the hand and finger in order to reduce errors during acquisitions and adopting an instrumental gold standard for measuring spasticity, may provide more insight into the validity of these measurements.

## Data availability statement

The raw data supporting the conclusions of this article will be made available by the authors, without undue reservation.

## Ethics statement

The studies involving human participants were reviewed and approved by the Comité de Ética de la Investigación con medicamentos del Hospital Universitario Severo Ochoa. The patients/participants provided their written informed consent to participate in this study.

## Author contributions

RU, AM, and JT: conceptualization. RU, AM, and CS-M: data curation and formal analysis. RU and AG: methodology. RU, AG, AO, and NL: investigation. JT: project administration. AM: software. AO and JT: supervision. RU, AM, AP, MB, FC, FL, AO, and JT: writing. RU, AG-M, FL, and JT: critical revisions. All authors contributed to the article and approved the submitted version.
